# Asthma and depression: a pragmatic review of the literature and recommendations for future research

**DOI:** 10.1186/1745-0179-1-18

**Published:** 2005-09-27

**Authors:** Melissa Opolski, Ian Wilson

**Affiliations:** 1 Department of General Practice, University of Adelaide SA 5005, Australia

**Keywords:** asthma, depression, depressive disorder, research

## Abstract

**Background:**

Although the association between asthma and psychosocial factors has long been recognised, it is only in the last decade that the impact of coexisting asthma and depression has become the focus of considerable research interest. However, the findings so far have been confusing and often contradictory. This paper sets out a methodical review and appraisal of the literature to date, including suggestions for future research.

**Method:**

PubMed and PsycINFO databases were used to search for English-language articles relating to asthma and depression research. The resulting articles were then reviewed and summarised, creating a report that was used to develop research recommendations.

**Results:**

The main findings from this review included: (a) results are mixed as to whether persons with asthma are more likely to be depressed than those without asthma; (b) asthma and depression may have an 'additive' adverse effect on the normal asthma-related quality of life reductions; (c) subjective measures of asthma severity may be more strongly related to depression than objective measures; (d) specific asthma symptoms appear to be linked to depression; (e) sadness and depression can produce respiratory effects consistent with asthma exacerbations; (f) depression appears to be negatively related to asthma treatment compliance; (g) corticosteroid use in asthma treatment has been associated with depression, though it is unclear how common this problem is in real life; (h) interventions that address the physical, psychological, and social consequences of asthma are likely to lead to the most successful treatment outcomes; (i) treating the depression of individuals with asthma is likely to minimise the negative effects of the coexistence; and (j) a number of common methodological problems were observed in the literature.

**Recommendations:**

There is a large amount of research yet to be undertaken to clarify issues around asthma and depression, with the overdue next step being to design integrated treatment approaches, and carry out large-scale prospective studies to determine the impact of using such approaches to treat individuals with depression and asthma. Such studies will be the only way in which some fundamental questions about the development and coexistence of these two conditions will be answered.

## 

Although the pathophysiology of asthma is clearly defined, the psychological influences are currently less well understood [1]. Depression is one of the most prevalent psychological problems in allergic patients [2], though it is often regarded as a somewhat 'natural reaction' to the diagnosis of a significant illness such as asthma [3-5]. However, while sadness and mild depression may be considered a fairly normal response to a diagnosis of chronic illness, more severe, chronic depression can lead to serious consequences for persons with asthma [4].

## Research history

The association between asthma and psychological factors has been recognised for centuries [6]. Asthma has long been considered a psychosomatic disease, and during the 1930s–50s, was even known as one of the 'holy seven' psychosomatic illnesses [7]. At that time, psychoanalytic theories described the aetiology of asthma as psychological [8], with treatment often primarily involving psychoanalysis and other 'talking cures' [9]. As the asthmatic wheeze was interpreted as the child's suppressed cry for his or her mother, psychoanalysts viewed the treatment of depression as especially important for individuals with asthma [10].

During the early 1970s, as the understanding of the aetiology and pathophysiology of asthma improved [11,12], the emphasis on psychological issues decreased. There were, however, increases in asthma-related morbidity and mortality in many first-world countries [13], which were eventually attributed to the inappropriate use of β-agonists without corticosteroids. This issue led to a resurrection of psychosocial research relating to non-medical factors in asthma, involving variables such as poor family functioning, poor doctor-patient relationships, and social difficulties [11]. In the 1980s–1990s the growing interest in psychosocial variables also led to a number of studies being conducted into the links between asthma and anxiety and variables such as 'negative affect' or 'psychological distress', and in the late 1990s the connections between asthma and depression came to the research forefront, as the potential importance of this relationship came to be recognised. Today, as a result of years of biopsychosocial research, asthma is considered a disease of the pulmonary system with genetic and allergic origins that is significantly affected by psychosocial factors.

## Asthma, depression, and quality of life

It is well documented that individuals with asthma tend to experience reduced health-related quality of life (HRQOL) [14], and although HRQOL tends to be lower for individuals with severe asthma, the effect on those with more mild asthma can also be considerable [15,16]. Asthma of any severity may lead to reductions in each of the physical, psychological, and social domains of HRQOL [17], with most people with asthma reporting some restriction on their life and having poorer health status than individuals without asthma [17,18]. A large population-based study by Ford et al [14] demonstrated that individuals with asthma had significantly lower HRQOL than those who had never had asthma, and also experienced an average of 10 days per month of impaired physical or mental health, almost double that of those who had never had asthma.

The Medical Outcomes Study demonstrated that depression and chronic disease often have 'additive' adverse effects on wellbeing [19,20], and there is evidence to support this finding for asthma and depression. People with coexisting asthma and depression have significantly lower overall HRQOL, and also experience lower physical and mental health functioning than those with similar asthma activity but fewer depressive symptoms [21-23]. These findings of reduced asthma-related quality of life are similar to those found in studies of other chronic medical conditions, where a history of depression has also been associated with poorer functional health status [19,20,24,25].

## Are people with asthma more likely to be depressed?

Investigations of the prevalence of depression in asthma have reported findings ranging from 1% to 45% of individuals with asthma also suffering from depression or depressive symptoms [22,23,26-31]. Unfortunately, this wide variation in the reported rates makes it difficult to draw conclusions, but most authors suggest an increased prevalence of depression in patients with asthma.

Various studies have investigated whether individuals with asthma are more likely to be depressed than those without asthma, and while a number of studies have supported this hypothesis [5,32-36], other investigators [1,27] have not found such evidence. In response to the differing findings in regards to this issue, Adams [37] theorised that differing sampling techniques may have been related to the discrepancies between findings.

## Asthma severity and depression

While it seems logical that having more severe asthma would be associated with an increased risk of depression, studies have reported somewhat mixed results. In investigations utilising objective measures of asthma severity (i.e. clinician diagnoses, airway reactivity testing), Mrazek [34] found that more severe asthma was associated with increased depressive symptoms; however, studies by Afari et al [21] and Janson et al [27] did not find this association. Thus, the evidence so far does not strongly support a direct relationship between objective asthma severity and depression. However, two studies that used patients' *subjective *ratings of their own asthma severity found significant relationships between perceived asthma severity and depressive symptoms [5,38].

Taken as a whole, these investigations seem to suggest that subjective measures of asthma severity may be more strongly related to depression than objective severity measures. One potential explanation for this possibility is that the individual's perception of their own asthma severity may be more important than the 'real' severity of the individual's asthma in determining whether or not asthma leads to the person becoming depressed.

## Specific asthma symptoms and depression

A small number of studies have investigated the links between specific asthma symptoms and depression. One of these, a population study by Goldney et al [39], examined the relationship between depression (as measured by the Prime-MD) and a number of symptoms known to be related to asthma severity, and found that dyspnoea, wakening at night, and morning symptoms were particularly strongly associated with depression. Another study by Janson et al [27] found significant positive correlations between depression (as determined by the Hospital Anxiety and Depression Scale (HADS)) and various, but not all, symptoms of asthma.

However, while specific symptoms of asthma may be linked to depression, it is also possible that the assessment of depression in the afore-mentioned studies could have been inaccurate due to the misinterpretation of asthma symptoms as symptoms of depression. Alternatively, it is possible that the findings and their interpretation are accurate, and certain symptoms of asthma may be related to depression due to their effect on the individual's quality of life [39]. The finding of Goldney et al [39] relating asthma symptoms to significant decrement in the quality of life also leads to the question of whether experiencing certain specific asthma symptoms might lead to person with asthma becoming depressed, rather than depression resulting from simply 'having' asthma.

## Can depression have a direct effect on pulmonary functioning?

The influence of emotional states on pulmonary function in asthma has been studied extensively [40]. Recent findings indicate that airways are reactive to psychological states, with these reactions causing changes consistent with greater airway instability and asthma exacerbations [41]. Consistent with this finding, personal retrospective accounts of asthma exacerbations have also suggested that changes in emotional states often result in asthma exacerbations [42].

In the laboratory, Ritz et al [43] found increased total respiratory resistance in subjects with asthma (but not those without asthma) following exposure to depressing stimuli. Krommydas et al [44] reported that individuals with asthma and symptoms of depression (measured by the Personal Disturbance Scale (DSSI/sAD)) had significantly lower FEV1% than individuals with asthma who showed no symptoms of depression. It is not clear from this study, however, whether the results are due to the depression or the depression is due to the reduced lung function.

Further, a study of overall physical vulnerability to asthma by Miller and Wood [45] suggested that depressive emotions in children (evoked whilst viewing selected scenes from the film 'ET: The Extra Terrestrial') may be associated with greater cholinergic influence and instability of oxygen saturation, consistent with poorer airway function in asthma. In contrast, happiness appeared to be related to effects that were more likely to relieve airway constriction.

Ritz and Steptoe [40] conducted a study consisting of both laboratory and field measurements, and demonstrated a consistency between both situations. From the field measurements, episodes of strong positive and negative mood were compared with records of relatively neutral emotional state, with the results showing that for persons with asthma (but not those in the non-asthmatic control group), negative affect, and to a lesser degree, positive affect, were associated with a reduction in FEV1%. These findings of reduced FEV1% during negative mood in the 'real life' component of the study were also consistent with and significantly related to increases in respiratory resistance during the viewing of a depressing film in the study's laboratory component.

Overall, the research on affect and pulmonary function indicates that depressed or sad mood, even when only short-lived and mild, can produce respiratory effects that are consistent with heightened airway vulnerability or asthma exacerbations. Further investigations are needed to establish whether this effect commonly occurs in the everyday lives of persons with asthma.

## Depression, asthma self-management, and treatment compliance

Self-management of asthma can be challenging, due to the often complicated and onerous nature of treatment [41,46]. The individual with asthma may need to avoid allergens; take preventive medication regularly; and decide when reliever medications are required and whether further medical assistance should be sought [41,46]. Further, the potential implications of poor management and reduced compliance are serious, with consistently negative effects for individuals with asthma being reported, including increased morbidity [47] and mortality [48,49].

Depression, which has negative effects on cognitive functioning, energy, and motivation [41], has been identified as one factor which may decrease the effectiveness of asthma self-management and compliance. A meta-analysis by DiMatteo et al [50] revealed that patients with a chronic disease and depression were three times more likely to be noncompliant with medical treatment than non-depressed patients. Bosley et al [51] found that individuals with asthma who were classified as 'noncompliant' (took less than 70% of their prescribed doses of inhaled medication) had significantly higher depression scores (measured on the HADS) than those who were compliant, and Cluley and Cochrane [11] reported that in their investigation of a group of 19 people with asthma who had been diagnosed with depression (as assessed by both the HADS and the Structured Clinical Interview for the DSM-III-R Patient Edition (SCID-P)), only one was adherent to their medication regime.

Depression might interfere with asthma treatment compliance and self-management via several pathways: firstly, depression-related hopelessness may lead to a patient seeing little point in taking their medication as instructed [52]; depression can also result in isolation from family and friends who could offer the support that has often been noted as important for compliance [52]; and finally, depression may also be associated with declines in areas of cognitive functioning (such as problem solving, complex task performance, concentration, attention span, and memory) that are vital for compliance with treatment recommendations [37,53-55].

Unfortunately, the correlational studies mentioned here cannot determine whether depression causes reduced compliance or vice-versa, whether there are mediating factors in the relationship, or whether the relationship between depression and compliance may be bi-directional [50]. DiMatteo et al [50] have also hypothesised the possibility of a 'feedback loop', in which depression leads to treatment non-compliance, non-compliance further exacerbates asthma, asthma exacerbations lead to increased depression, and so on, resulting in a cycle of ever-worsening outcomes for the individual.

## Corticosteroid use and depression

Corticosteroid use has also been hypothesised as a link between asthma and depression [18,46,56]. The creation of more potent inhaled corticosteroids (ICS), together with more efficient delivery systems, has greatly increased the use of ICS in the last 20 years [18]. While these changes have had positive effects on overall health, they have also been related to adverse side effects [57].

Although early reports of a possible relationship between asthma medications and depression were rare, and often anecdotal, recent studies in this area seem to suggest a stronger link than first believed. For example, an investigation by Patten and Lavorato [58] found that corticosteroid use was significantly associated with a 'syndrome resembling major depression' (measured by the Composite International Diagnostic Interview Short-Form (CIDI-SF)), and in a study by Patten [59] of 73,402 community members, it was found that among individuals taking corticosteroids (current or within the past month), the prevalence of major depression (11.1%; also measured by the CIDI-SF) was significantly higher than for those who had not taken steroids (4.1%). Perceived health status was not a confounder in this study. In a smaller outpatient study (N = 50), Bonala et al [18] found high-dose ICS usage was associated with positive effects in relation to pulmonary functioning and 'physical' quality of life, but was inversely related to 'mental' quality of life.

There appear to be a number of gaps and problems in the literature to date on ICS and depression in asthma. For example, some studies seem to have overlooked the possibility that the illness for which the corticosteroid was being taken may have been responsible for the increased levels of depression, instead of the medication. Also, it is unclear if depression related to corticosteroid use is actually a common problem. As mentioned, earlier accounts of this relationship were infrequent, and it may be that as ICS have become more potent and prescribed, the problem has increased exponentially. Another possible explanation for increased recognition of depression in persons using ICS for asthma treatment may simply be that medical practitioners have become more adept in recognising depression.

## A 'feedback loop' of depression and asthma?

A number of researchers have suggested that a so-called 'feedback loop' may exist between asthma and depression [42,45]. Lehrer et al [42] noted that negative emotion (such as depression) often experienced by people with asthma may be as much a result of having asthma, as it is a cause of it, and that this bidirectional association may lead to a continual cycle of asthma and depression, resulting in ever-worsening physical and mental health. DiMatteo et al [50] noted the possibility that non-compliance with medical treatment might also be a component in this cycle.

## Clinical implications: a 'whole person' approach to treatment

As discussed above, the coexistence of asthma and depression has been linked to a number of negative effects on both the physical and mental health of the individual. These adverse effects have led several researchers to propose that the most successful treatment is likely to require an integrated treatment approach, using interventions to address the physical, psychological, and social consequences of asthma [45,60].

One suggested treatment combination has been to treat depression and asthma together using antidepressants with anticholinergic properties, such as tricyclic antidepressant medication [4,45,60]. However, some who have advocated the use of antidepressant medications have also warned that antidepressants alone may not be the entire answer for a chronic disease such as asthma, because people with asthma need to be able to "successfully grieve over physical losses, combat changes in self-esteem, and overcome the social isolation that illness can cause" [[60], p.296]. For these reasons, counselling and psychosocial aspects in treatment are likely to have a very important role in successful asthma treatment, and cognitive-behavioural therapy (CBT) and group and individual counselling are already being combined with asthma self-management information to try to improve health outcomes for asthma sufferers [61].

It is hoped that by treating depression in asthma, the negative effects of this coexistence can be minimised [33,51]. While treating depression may increase adherence and lead to more effective asthma self-management, decrease asthma symptoms, and possibly even decrease asthma-related mortality [11,44,61-63], at a minimum, treating depression is likely to dramatically improve the HRQOL of individuals with asthma [64].

To date, only one study is believed to have examined the impact of treating depression in asthma. Grover et al [65] recruited a sample of 10 asthma out-patients, with participants sequentially allotted to either the experimental group (CBT and standard pharmacotherapy for asthma) or control group (standard pharmacotherapy only). CBT in the experimental group consisted of 15 individual sessions involving asthma education, muscle relaxation techniques, behavioural techniques, cognitive restructuring, and coping skills. Following the full course of therapy and medication, the experimental group had significant decreases in asthma symptoms, anxiety, and depression (measured by the Beck Depression Index), and a significant increase in quality of life, while the control group did not show any significant changes. Although these results are promising, further investigation into the effects of employing combined treatment approaches is obviously necessary.

## Methodological issues in the literature

A number of problematic methodological issues were observed in the studies examined in this review, many of which limited interpretations of the findings. Firstly, the majority of the reviewed studies were cross-sectional and retrospective in design, meaning that neither directionality nor causality could be reliably inferred from the results [22,28,37,42,50,66]. Instead, many researchers hypothesised causality and relationship direction in their own findings based on the conclusions of other studies.

The different measurement instruments used and names given to the constructs of depression examined in the studies, ('depression', 'depressive symptoms', 'major depression', 'a syndrome resembling major depression', etc) also made it difficult both to understand what was actually being measured and investigated, and to compare results and interpretations between studies. Comparatively few studies used standardised diagnostic instruments such as the DSM and SCID, or standardised clinical rating scales such as the Hamilton Rating Scale for Depression, the consistent use of which would make interpretation and cross-study comparisons more straightforward. The problems of unclear definitions of depression and lack of standardised assessment measures were also noted by Rodin and Voshart [cited in [67]] as issues hindering progress on research in the medically ill.

Because asthma and depression share a number of symptoms, there is also potential for the use of self-report measures in studies to result in inaccurate diagnoses of depression or depressive symptoms [23,37,68]. Sherwood Brown et al [23] noted that on some measures, secondary symptoms of asthma such as decreased sleep or fatigue may elevate scores on measures of depression, even if these symptoms are actually unrelated to psychological problems. This issue will require careful selection of instruments to measure depression.

Finally, most samples utilised in studies investigating asthma and depression have been drawn from in-patient or outpatient samples, with very few population or primary care studies conducted thus far. Although these studies have been instrumental in highlighting links between asthma and depression, the generalisability of these findings to the broader asthma population is not yet well understood [39].

## Future research directions

There are many potential research avenues to consider in regards to the coexistence of asthma and depression. Firstly, further investigation of the various links considered in this review (especially those explored in studies that may have been limited by methodological issues) would serve to provide a clearer understanding of the relationships between asthma and depression, and the potential repercussions of this association.

Future research should also consider 'big picture' studies, in which a number of different variables such as medication compliance, asthma self-management, quality of life, and lung function might all be examined in one investigation. These studies are likely to be particularly vital in aiding our understanding of possible 'feedback loops' of asthma and depression, more fully exploring how asthma and depression coexist, and may be valuable in determining how best to reduce or eliminate the negative effects of this coexistence.

Researchers have been suggesting for some time that the most important next step in asthma and depression research is to investigate the effects of treating the depression of persons with asthma. However, as previously mentioned, only one small study appears to have attempted this so far [65]. The first step in this new research phase would be to develop effective combinations of pharmacological, psychological, and/or psychosocial interventions as an integrated treatment program for people with asthma and depression. The most sought after knowledge, however, would result from studies using these integrated programs to actually treat the depression of individuals with asthma, and assessing the effects of this treatment on variables such as the depression itself, compliance, self-management, HRQOL, pulmonary function, asthma symptom exacerbations, overall asthma severity, and asthma-related mortality. Large scale studies of this type would be complex to set up, expensive to carry out, and require long-term commitment to the research.

Although they are most likely to be targeted toward patients already suffering from both asthma and depression, integrated treatment programs may also be valuable from a prevention focus, working to inhibit the development of depression and the negative psychological and physical health effects that may follow. Large scale, long-term research also needs to be carried out to examine this important possibility.

While a great deal of investigation still needs to be carried out, the move into this new phase of asthma and depression research has great potential to result in more effective ways of caring for people living with these coexisting illnesses, and significantly improve the lives of many individuals.

## Declarations

Ethical clearance was not required for this literature review. MO received a University of Adelaide Vacation Scholarship to conduct the literature review. The authors do not have any conflict of interests in relation to this study.
